# PET-MRI tracking of imaging-visible microencapsulated stem cells in immunocompetent rabbits

**DOI:** 10.1186/1532-429X-15-S1-M1

**Published:** 2013-01-30

**Authors:** Yingli Fu, Ronnie Mease, Ying Chen, Guan Wang, Dorota Kedziorek, Meiyappan Solaiyappan, Dara Kraitchman

**Affiliations:** 1 Radiology and Radiological Science, Johns Hopkins University, Baltimore, MD, USA; 2 Electrical and Computer Engineering, Johns Hopkins University, Baltimore, MD, USA

## Background

Exogenous stem cell therapy has shown benefits for treating peripheral arterial disease patients, who are not amenable for conventional revascularization therapy. Previously, we have demonstrated the ability of imaging-visible cell microencapsulation to overcome the challenges of poor cell retention/survival and difficulties with monitoring cell delivery success. However, *in vivo* cell viability cannot be assessed noninvasively. Here, we investigate the potential of PET-MRI tracking of ^19^F MRI-visible microencapsulated human mesenchymal stem cells (hMSCs) labeled with triple-fusion (TF) reporter gene in non-immunosuppressed rabbits.

## Methods

Bone marrow-derived hMSCs were stably transfected with a lentiviral vector encoding firefly luciferase, red fluorescence protein, and thymidine kinase. Alginate cell microencapsulation was performed using a modification to incorporate perfluorooctylbromide (PFOB). Bioluminescence imaging (BLI, Xenogen IVIS 2000) was acquired before and after cell encapsulation to assess *in vitro* cell viability. Rabbits received either intramuscular injection of PFOB-encapsulated TF-hMSCs in the medial thigh followed by intravenous administration of [18F] 9-[4-fluoro-3-(hydroxymethy) butyl] guanine ([18F]-FHBG) (n=7, 1.7±0.7 mCi), or PFOB-encapsulated TF-hMSCs that were pre-incubated with [18F]-FHBG (n=3, 55±2 µCi). Dynamic PET imaging (Siemens HRRT CPS Innovation) was acquired immediately or 60 min after transplantation for 30-90 min. Proton (3D GRE, TR/TE=15/5.45 ms, FOV=186x230 mm, voxel size=0.45x0.45x1.5mm) and 19F MRIs (TrueFISP, Siemens Tim Trio, TR/TE=4.1/2.0 ms, 32 averages, FOV=250x250 mm, image matrix=192x192, BW=1002 Hz/pixel, voxel size= 1.3x1.3x1.25 mm) were obtained 1-2 days after delivery. PET images were fused with 1H/19F MR to identify the location of transplanted cells. Follow-up PET imaging was repeated within 1-14 days with intravenous or ultrasound-guided intramuscular (0.7±0.3 mCi/thigh) of administration of [18F]-FHBG. Follow-up 19F MRIs were acquired 1-2 days after PET imaging. BLI was performed 2 weeks after delivery.

## Results

**Figure 1:**
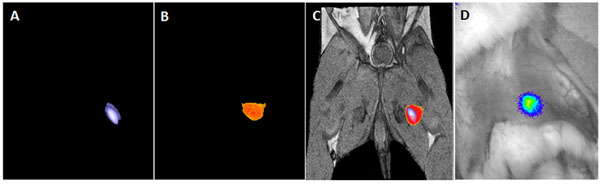
(A) ^19^F MR image of PFOB Caps containing TF-hMSCs in a the rabbit thigh; (B) PET image of PFOB Caps in the same rabbit; (C) Fusion of ^19^F MR image (blue) and PET image (red) with anatomical ^1^H MR showing the concordance “hot spot” and the location of PFOB Caps injection site; (D) Bioluminescence imaging of the rabbit revealing highly viable encapsulated TF-hMSCs 2 weeks after delivery.

*In vitro* hMSC viability and transgene expression were not affected by encapsulation or [18F]-FHBG incubation as determined by BLI and live/dead cell staining (91±6%). All PFOB cap injections were identified on ^19^F MRI *in vivo* (Figure 1A). Using PET, PFOB microcapsule injection site in rabbit thigh was identified as "hot spot" (Figure 1B) and showed high concordance to the MRI "hot spot" (Figure 1C). No significant volume change of PFOB Caps was observed on ^19^F MRI over 2 weeks. BLI demonstrated viable xenogeneic TF-hMSCs in PFOB microcapsules 2 week post delivery (Figure 1D).

## Conclusions

We demonstrate xenogeneic MSC delivery in non-immunosuppressed large animals using novel MRI-visible microencapsulation and reporter gene labeling. PFOB microencapsulation of TF-hMSCs enables cell tracking and viability assessment using clinical PET-MRI.

## Funding

Funding support was provided by NIH R21/R33-HL89029 & MD-SCRFII-039

